# Squamous cell carcinoma of the external ear

**DOI:** 10.11604/pamj.2023.45.83.38634

**Published:** 2023-06-13

**Authors:** Xinrong Chen, Jiexia Qiu

**Affiliations:** 1 Zhuji People's Hospital of Zhejiang Province, Shaoxing, 311800, China

**Keywords:** Squamous cell carcinoma, external ear

## Image in medicine

Cutaneous squamous cell carcinoma can occur on any surface of the skin, including the head, neck, trunk, limbs, and oral mucosa, the diagnosis of which is mainly made upon histopathology. A 72-year-old patient suffering from progressive enlargement and pain in his left external ear for more than 5 years presented in the outpatient department. A dermatological examination revealed that his left external ear had been dilated by an area of about 4 x 3cm. Diagnosed as squamous cell carcinoma through histologic examination, he received surgical resection without recurrence.

**Figure 1:**
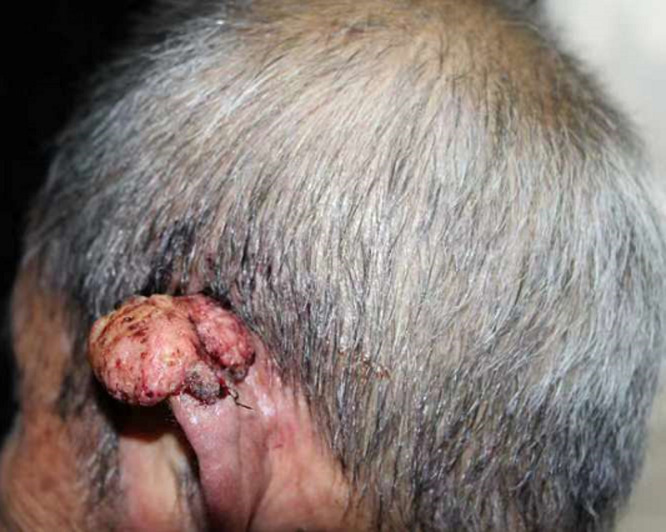
the left external ear was dilating with a diameter of about 4 x 3cm

